# Investigating the Impact of Item Parameter Drift for Item Response Theory Models with Mixture Distributions

**DOI:** 10.3389/fpsyg.2016.00255

**Published:** 2016-02-24

**Authors:** Yoon Soo Park, Young-Sun Lee, Kuan Xing

**Affiliations:** ^1^Department of Medical Education, College of Medicine, University of Illinois at ChicagoChicago, IL, USA; ^2^Department of Human Development, Teachers College, Columbia UniversityNew York, NY, USA; ^3^Department of Educational Psychology, College of Education, University of Illinois at ChicagoChicago, IL, USA

**Keywords:** item parameter drift, item response theory, mixture IRT, TIMSS, differential item functioning

## Abstract

This study investigates the impact of item parameter drift (IPD) on parameter and ability estimation when the underlying measurement model fits a mixture distribution, thereby violating the item invariance property of unidimensional item response theory (IRT) models. An empirical study was conducted to demonstrate the occurrence of both IPD and an underlying mixture distribution using real-world data. Twenty-one trended anchor items from the 1999, 2003, and 2007 administrations of Trends in International Mathematics and Science Study (TIMSS) were analyzed using unidimensional and mixture IRT models. TIMSS treats trended anchor items as invariant over testing administrations and uses pre-calibrated item parameters based on unidimensional IRT. However, empirical results showed evidence of two latent subgroups with IPD. Results also showed changes in the distribution of examinee ability between latent classes over the three administrations. A simulation study was conducted to examine the impact of IPD on the estimation of ability and item parameters, when data have underlying mixture distributions. Simulations used data generated from a mixture IRT model and estimated using unidimensional IRT. Results showed that data reflecting IPD using mixture IRT model led to IPD in the unidimensional IRT model. Changes in the distribution of examinee ability also affected item parameters. Moreover, drift with respect to item discrimination and distribution of examinee ability affected estimates of examinee ability. These findings demonstrate the need to caution and evaluate IPD using a mixture IRT framework to understand its effects on item parameters and examinee ability.

## Introduction

The invariance of item parameters calibrated from the same population is an important property of item response theory (IRT) models, which extends to estimates measured at different occasions (Lord, 1980; Hambleton and Swaminathan, 1985; Hambleton et al., 1991; Baker and Kim, 2004). For anchor items used to link and scale scores between different tests, the invariance property of item parameters becomes a necessary condition, because without it, ability scores cannot be comparable–in IRT, the probability of getting an item correct is a function of the examinee's ability and item parameters. Item parameters may change over time due to factors other than sampling error. When this occurs, items can be considered to be easier or less discriminating than their true estimates. In general, deviations in item parameters from the true value to its successive testing administrations are known as *item parameter drift* (IPD; Goldstein, 1983; Wells et al., 2002, 2014). IPD occurs when invariance no longer holds, and there is a differential change in item parameters over time.

There are various studies that have examined the cause of IPD. As suggested in Mislevy (1982), Goldstein (1983), and Bock et al. (1988), one possible source of drift may be changes in the curriculum. For example, the Trends in International Mathematics and Science Study (TIMSS) was designed to assess students' knowledge of curricular topics in mathematics and science (i.e., reproduction of knowledge; e.g., Hutchison and Schagen, 2007; Olson et al., 2009). Because TIMSS evaluates performance of curriculum attainment, the invariance property of its anchor items serves as an important property for scaling scores across different test administrations.

The study of IPD is related to differential item functioning (DIF) in that both detect item bias and are rooted in measurement invariance. However, the difference between IPD and DIF rests in the notion that the latter examines differences between manifest groups (e.g., gender, race, income), while the former is between testing occasions (Rupp and Zumbo, 2006). As a method for detecting DIF in latent subpopulations, mixture IRT models base their consideration from mixture distribution models (MDM) that rejects the homogeneity of the observed data. Mixture IRT models consider a mixture of latent subpopulations to constitute the sample (Everitt and Hand, 1981; Titterington et al., 1985). In other words, a particular set of item parameters are no longer valid for the entire sample, and unique model parameters are estimated for each homogeneous subpopulation (i.e., latent classes).

The motivation for this study comes from large-scale testing programs, such as TIMSS, National Assessment of Educational Progress (NAEP; Mislevy et al., 1992), and the Programme for International Student Assessment (PISA; OECD, 2014) that use unidimensional IRT models to calibrate item parameters and scale examinee performance on multiple test cycles. In these testing programs, item invariance is checked by testing for DIF at baseline (first test administration); then, on successive test administrations, tests for IPD are conducted to ensure item parameters are consistent over time. However, these methods fail to check for the presence of latent subgroups that may exist at baseline or that may appear on successive test administrations. In fact, prior studies have largely ignored the impact of calibrating item parameters using unidimensional IRT models, even when latent subgroups may exist in the examinee population. Moreover, tests for item invariance are generally limited to potential subgroup differences for manifest variables using DIF analyses. The presence of latent subgroups violates the item invariance property of unidimensional IRT models and therefore can bias the inferences resulting from successive item calibrations (DeMars and Lau, 2011). As such, this paper serves as a cautionary note to investigate the magnitude of potential bias that may occur as a result of ignoring to check for latent subgroups and the successive bias that may also result in subsequent test administrations.

This study examines the impact of IPD on parameter and ability estimates when the underlying measurement model holds mixture distributional properties, thereby violating the invariance assumption for IRT models. Evidence of latent subgroups in large-scale tests, such as TIMSS has been shown previously (e.g., Choi et al., 2015). Yet, studies have generally focused on the effect of IPD on examinee ability and on parameter estimates (e.g., Wells et al., 2002; Miller et al., 2005; Babcock and Albano, 2012), noting that substantial drift can result in significant bias. These studies have not specifically evaluated the invariance assumption by testing for mixture distributions—that is, if the underlying measurement models lack invariance in parameter estimates in the baseline IRT estimates due to latent subgroup differences, then parameters and ability estimates can be biased, beyond the effect that IPD may solely have. As such, it becomes important to investigate the impact of IPD, considering implications when a mixture model better fits the underlying data. Findings from this study aim to underscore the importance of testing for invariance of IRT parameters from mixture distributions, particularly when operational uses of the measurement model are for equating or linking purposes.

This study is divided into two sections, (1) an analysis of real-world data using the TIMSS to demonstrate violation of item invariance through mixture IRT models and (2) a simulation study that uses the empirical results for further analysis. Results from the empirical study are used to provide specific conditions in the simulation study to examine the effect that mixture IRT models can have on item parameters and ability estimates. In the first phase of this study, TIMSS mathematics data were analyzed to examine the prevalence of IPD using 21 trended anchor items from the 1999, 2003, and 2007 administrations of TIMSS; these items were used to link and scale the ability estimates between the tests. However, rather than employing traditional methods of IPD (e.g., studying changes in item parameter estimates using unidimensional IRT models), this study examines changes in item parameter estimates using a mixture IRT model. Although the assumption of invariance may be satisfied in the unidimensional case, IPD may exist when item responses are modeled under the mixture IRT model.

In the second phase of this study, the effect of fitting data generated from mixture IRT model with IPD using a unidimensional IRT model is examined. This simulation study was motivated in that anchor items used in TIMSS assume item invariance using unidimensional IRT models. However, latent subgroups with IPD may exist. This study focuses on this case and investigates the impact of item and ability estimates. Estimated item parameters from TIMSS were used as true generating values to simulate data using a mixture IRT model for a realistic simulation. Conditions that examine the effect of IPD using changes in item difficulty and discrimination as well as the distribution of examinee ability were considered. Furthermore, patterns of IPD that affect the classification accuracy were also investigated. Results from this study aim to caution researchers and practitioners on consequences of ignoring latent subgroups, particularly when the measurement model relies on such invariance assumptions.

## Mixture item response theory models

Latent subgroups can be detected and estimated using mixture IRT models, which simultaneously defines a discrete mixture model for the item responses to unmix data into homogeneous subpopulations and applies estimation methods to derive model parameters for each latent class identified (Rost, 1990). Applications of mixture IRT models have been prevalent in DIF analyses as alternatives to traditional methods that test IRT model invariance across a priori known grouping information using manifest criteria such as gender, ethnicity, and age (Schmitt et al., 1993). As noted in Ackerman (1992), DIF occurs with the presence of a nuisance dimension that conflicts with the intended ability to be measured; therefore, manifest groupings may not always sustain the characteristics that classify such differences. An advantage of mixture IRT models is its ability to sort a priori unknown grouping based on examinees' response patterns and do not rely on information about the group. Furthermore, mixture IRT models can be used to examine factors that contribute to the DIF of examinees. Rost et al. (1997) analyzed personality scales to conduct such analysis. More recently, studies have been conducted to evaluate the cause of DIF in mathematics items (e.g., Cohen and Bolt, 2005; Cohen et al., 2005). Other applications have been explored using the mixture Rasch model with ordinal constraints within the context of testing speededness and scale stability (Bolt et al., 2002; Wollack et al., 2003).

In a mixture 3PL IRT model, the probability that examinee *i* answers item *j* correctly is formulated as follows:

$$\begin{array}{l} P(Y_{ij}=1|\theta_{ig},g,\alpha_{jg},\beta_{jg},c_{jg})\\ \;=c_{jg}+\frac{1-c_{jg}}{1\;+\;\exp[-1.702\alpha_{jg}(\theta_{ig}\;-\;\beta_{jg})]}, \end{array}$$

where θ_*ig*_ is examinee *i* 's ability in a latent group *g*, and α_*jg*_, β_*jg*_, and *c*_*jg*_ are item discrimination, difficulty, and guessing parameters, respectively for the latent group *g* (von Davier and Rost, 1995; Maij-de Meij et al., 2008). Restrictions on the parameters such as setting the guessing parameter to 0 or restricting parameter to be equal can change the model above to the 2PL or the 1PL model, respectively. When there are *G* latent groups, such that *G* ≥ 2, the unconditional probability that examinee *i* answers item *j* correctly becomes the following:

$$\begin{array}{l} P(Y_{ij}=1|\theta_{ig},\alpha_{jg},\beta_{jg},c_{jg})\\ \;=\sum_{l\;=\;1}^{G}\left\{\pi_{l}\left[c_{jg}+\frac{1-c_{ij}}{1+\exp\left[-1.702\alpha_{jg}\left(\theta_{jg}-\beta_{jg}\right)\right]}\right]\right\},s.t.\\ \;\sum_{g}\pi_{g}=1,0<\pi_{g}<1 \end{array}$$

The latent class membership variable is assumed to be parameterized as a multinomial random variable, since it only takes nonnegative integers. If there are only two latent classes, it can be parameterized as a Bernoulli random variable as follows:

$$\begin{array}{l} P\left(\Phi \right)=categorical\left(\pi_{g}\right)\Rightarrow P\left(\Phi \right)=Bernoulli\left(\pi_{1}\right). \end{array}$$

Here, π_*g*_ is the probability of membership.

Estimation for mixture IRT models has been examined by Rost (1990), Samuelsen (2008), and Lu and Jiao (2009), to investigate the recovery of items parameters and subgroup classification for varying conditions, such as number of items, sample size, mixing proportions, and differences in mean ability. These studies have found that parameters were recovered and latent classes were accurately classified. Moreover, Li et al. (2009) conducted an expansion of these previous studies and also found parameters to be recovered, particularly when the number of subgroups was lower, sample size greater, and there were more items. These prior studies have used maximum likelihood for mixture 1PL model (mixed Rasch model; Rost, 1990) and MCMC for mixture 2PL and 3PL models for estimation. For 1PL models, the ability to use conditional maximum likelihood facilitated estimation; however, for 2PL and 3PL models, Bayesian estimation using Markov chain Monte Carlo (MCMC) was used as it allowed greater flexibility to specify parameters to the model. In mixture models, label switching (permutation of class labels) can occur, because the likelihood function remains the same despite model parameters that can vary for different classes (Cho et al., 2012). The occurrence of label switching can be checked manually by examining the class sizes in each MCMC iteration or by specifying constraints on parameters such that the mixing proportions have ordering restrictions (McLachlan and Peel, 2000).

## Study 1: a real data example – TIMSS 1999, 2003, and 2007

### Trends in international mathematics and science study (TIMSS)

The TIMSS has been conducted since 1995 and is administered on a four-year cycle. Researchers have analyzed trends in international mathematics achievement based on overall performance of students using both the TIMSS and the PISA (Baucal et al., 2006; Black and Wiliam, 2007; Takayama, 2007). Although there are several empirical studies on IPD in the educational and psychological measurement literature (e.g., Skykes and Ito, 1993; Juve, 2004; Pleysier et al., 2005), there are only a few that have specifically examined IPD in international assessments. For example, Wu et al. (2006) conducted a comparison study between the U.S. and Singapore to detect IPD. They showed that using 23 trended anchor items from the 8th grade 1995, 1999, and 2003 administrations of TIMSS, there was no IPD. However, to date, there are far fewer studies that have examined IPD to check for measurement invariance using a mixture IRT framework, and none that have been applied to an assessment such as the TIMSS. In the 2007 TIMSS database, 21 trended items from 1999, 2003, and 2007 were released. These items were used in this study to examine IPD in real-world data.

The purpose of the real-world data example is to demonstrate the presence of latent subgroups and IPD using mixture IRT for large-scale testing data that assumes population homogeneity. The TIMSS calibrates item parameters based on the assumption that there are no latent subgroups. The presence of latent subgroups, in addition to IPD, would serve to show the practical application of this study and the importance of checking for latent subgroups, which violate the item invariance property of unidimensional IRT models. The impact of ignoring to check for latent subgroups is further investigated in the simulation study that follows this section.

### Methods

This study analyzed item responses of the U.S. 8th graders based on mathematics achievements from TIMSS 1999, 2003, and 2007. TIMSS partially releases its items, so that they can reuse unreleased items on subsequent testing cycles. Among 109 released items in TIMSS 2007, 21 items were also administered in 1999 and 2003 TIMSS mathematics for 8th graders. These items were well-balanced as they represented four content domains—Number, Algebra, Geometry, and Data & Chance. The number of students who took the 21 items varied between the testing cycles. In 1999, 2003, and 2007 TIMSS administrations, 2291, 4473, and 2124 students from the U.S. sample took the entire set of 21 items or selected subset of these items.

WinBUGS 1.4 (Lunn et al., 2005) was used to fit the IRT models using MCMC estimation from the Gibbs sampler algorithm; this procedure simulates a Markov chain to sample values for the parameters of the full conditional posterior distributions. The unidimensional 3PL IRT model and mixture 3PL IRT models were each fit separately for the three time points. The 3PL IRT models were used, because TIMSS calibrates its item parameters using the 3PL IRT model as described in the *TIMSS 2007 Technical Report* (Olson et al., 2009). Prior study by Choi et al. (2015) also used the mixture 3PL model to analyze the TIMSS 2007 data and found two latent subgroups.

The Bayesian method also requires the specification of prior distributions to derive the posterior distribution. As such, flat priors with large variance were used to specify the prior distributions. The choice of prior distributions was based on previous studies that have shown consistent estimates using mixture IRT models in both simulation studies and in large-scale testing contexts (e.g., Bolt et al., 2002; Cohen and Bolt, 2005; Cho and Cohen, 2010). The following prior distributions were used:

$$\begin{array}{l} \begin{array}{c} \alpha_{jg}\;\sim \;Normal\left(0,4\right)\\ \beta_{jg}\sim Normal\left(0,4\right)\\ c_{jg}\sim Beta\left(5,17\right) \end{array}\;\begin{array}{c} \theta_{i}\sim Normal\left(0,\tau^{\left(\theta \right)}\right)\\ \tau^{\left(\theta \right)}\sim Gamma\left(0.5,1\right)\\ \pi_{g}\sim Dirichlet\left(0.5,0.5\right) \end{array} \end{array}$$

The probability that a student correctly answered an item (i.e., *Y*_*ij*_ = 1) was modeled using the 3PL models such that *Y*_*ij*_ ~ *Bernoulli*(*p*_*ij*_). Bayesian versions of Akaike Information Criterion (AIC) and the Bayesian Information Criterion (BIC) statistics (Spiegelhalter et al., 2002) were calculated to compare the fit of the unidimensional 3PL IRT model to the two-class mixture 3PL IRT model for each of the three test administrations following recommendations for model selection based on Li et al. (2009). Since data were estimated using MCMC (Stephens, 2010), label switching was checked for each data iteration, by examining the class sizes (Cho et al., 2012).

### Results

The models were run with 10,000 samples and 4000 samples as burn-in; the sample autocorrelations and monitoring statistic of Gelman and Rubin (1992) were satisfied for the convergence diagnostic tests.

#### Item parameters: unidimensional 3PL IRT model

Table 1 shows the item parameter estimates of the 3PL IRT model. For the unidimensional 3PL IRT model, there was evidence of IPD for the discrimination and difficulty parameters; for the pseudo-guessing parameter, IPD was less than 0.03 on average. IPD was examined by calculating absolute deviance measures. The mean absolute deviations (bias) in item discrimination estimates between 1999 and 2003 and between 2003 and 2007 were 0.35 and 0.35, respectively, with an overall mean deviation of 0.25 across the three administrations. There were items with discrimination parameter changes of about 1. For example, for items 16 and 21, the discrimination decreased by 0.90 and 1.38, respectively. For item difficulty, the average deviation between 1999 and 2003 was 0.38; between 2003 and 2007, it was 0.50, with an overall mean absolute deviation of 0.51 between 1999 and 2007. Taking into account both decrease and increase in parameter estimates, there was no overall change in item discrimination; however, results showed that items were easier for students over the three administrations (overall change was 0.34). These results provide some evidence of IPD even in the unidimensional 3PL IRT model.

**Table 1:** **Item parameter estimates of the 21 trended anchor items in 1999, 2003, and 2007 TIMSS: Unidimensional 3PL IRT model**.

**Item**	**Discrimination (*****a*****)**			**Difficulty (*****b)***			**Lower Asymptote (*****c*****)**		
	**1999**	**2003**	**2007**	**1999**	**2003**	**2007**	**1999**	**2003**	**2007**
1	0.65 (0.01)	0.47 (0.01)	0.62 (0.01)	−1.35 (0.04)	−2.52 (0.05)	−2.03 (0.04)	0.19 (0.00)	0.22 (0.01)	0.22 (0.01)
2	1.26 (0.02)	0.92 (0.02)	0.98 (0.02)	−0.61 (0.02)	−0.81 (0.02)	−0.95 (0.02)	0.15 (0.00)	0.16 (0.00)	0.16 (0.00)
3	0.61 (0.01)	0.49 (0.01)	0.52 (0.01)	−0.58 (0.03)	−0.31 (0.04)	−0.26 (0.04)	0.19 (0.00)	0.26 (0.01)	0.21 (0.01)
4	0.91 (0.02)	0.90 (0.02)	1.00 (0.02)	1.56 (0.03)	1.51 (0.03)	1.25 (0.02)	0.14 (0.00)	0.18 (0.00)	0.19 (0.00)
5	2.13 (0.04)	1.46 (0.02)	2.25 (0.03)	1.18 (0.02)	1.19 (0.02)	1.17 (0.02)	0.03 (0.00)	0.03 (0.00)	0.04 (0.00)
6	0.53 (0.01)	0.42 (0.01)	0.59 (0.01)	−1.65 (0.05)	−1.15 (0.10)	−0.68 (0.05)	0.22 (0.01)	0.29 (0.01)	0.26 (0.01)
7	1.27 (0.02)	0.93 (0.02)	1.67 (0.03)	2.26 (0.05)	2.68 (0.04)	2.10 (0.03)	0.09 (0.00)	0.09 (0.00)	0.11 (0.00)
8	1.78 (0.03)	1.35 (0.02)	1.74 (0.03)	0.16 (0.01)	0.26 (0.01)	0.44 (0.01)	0.08 (0.00)	0.09 (0.00)	0.09 (0.00)
9	1.60 (0.03)	1.09 (0.02)	1.94 (0.04)	−0.98 (0.02)	−1.54 (0.03)	−0.87 (0.02)	0.20 (0.00)	0.15 (0.00)	0.18 (0.00)
10	0.93 (0.02)	0.62 (0.01)	0.77 (0.01)	−1.93 (0.04)	−2.52 (0.05)	−2.29 (0.04)	0.24 (0.01)	0.19 (0.00)	0.23 (0.00)
11	1.48 (0.03)	1.01 (0.02)	1.44 (0.03)	−0.97 (0.02)	−1.68 (0.03)	−1.08 (0.02)	0.21 (0.00)	0.16 (0.00)	0.19 (0.00)
12	0.78 (0.01)	0.64 (0.01)	1.21 (0.04)	1.64 (0.03)	2.12 (0.04)	1.52 (0.03)	0.10 (0.00)	0.15 (0.00)	0.19 (0.00)
13	1.11 (0.02)	0.99 (0.02)	1.37 (0.04)	1.33 (0.03)	1.51 (0.02)	1.18 (0.02)	0.10 (0.00)	0.07 (0.00)	0.13 (0.00)
14	0.96 (0.02)	0.80 (0.01)	0.75 (0.02)	1.18 (0.03)	0.95 (0.02)	0.62 (0.03)	0.26 (0.00)	0.27 (0.00)	0.23 (0.01)
15	0.74 (0.01)	0.66 (0.01)	0.42 (0.01)	1.07 (0.03)	1.16 (0.02)	−2.93 (0.06)	0.15 (0.00)	0.14 (0.00)	0.26 (0.00)
16	1.84 (0.04)	0.94 (0.01)	1.14 (0.02)	−1.12 (0.03)	−2.33 (0.04)	−2.30 (0.04)	0.30 (0.01)	0.22 (0.00)	0.23 (0.00)
17	1.18 (0.02)	1.04 (0.02)	1.02 (0.02)	1.97 (0.04)	1.80 (0.03)	2.08 (0.03)	0.02 (0.00)	0.02 (0.00)	0.04 (0.00)
18	1.72 (0.03)	1.40 (0.02)	1.77 (0.03)	1.69 (0.03)	1.92 (0.03)	1.52 (0.02)	0.01 (0.00)	0.02 (0.00)	0.03 (0.00)
19	2.06 (0.04)	1.49 (0.02)	1.93 (0.03)	1.87 (0.04)	2.18 (0.04)	1.82 (0.03)	0.01 (0.00)	0.01 (0.00)	0.02 (0.00)
20	1.24 (0.02)	1.18 (0.02)	1.81 (0.03)	1.62 (0.03)	1.77 (0.03)	1.42 (0.02)	0.06 (0.00)	0.05 (0.00)	0.07 (0.00)
21	2.77 (0.05)	1.39 (0.02)	1.94 (0.03)	0.75 (0.02)	0.43 (0.01)	0.30 (0.01)	0.26 (0.00)	0.20 (0.00)	0.11 (0.00)

*Estimated using MCMC with 10,000 samples and 4000 samples burn-in; values in parenthesis represent MC Error*.

#### Item parameters: two-class mixture 3PL IRT model

**Figure 1:**
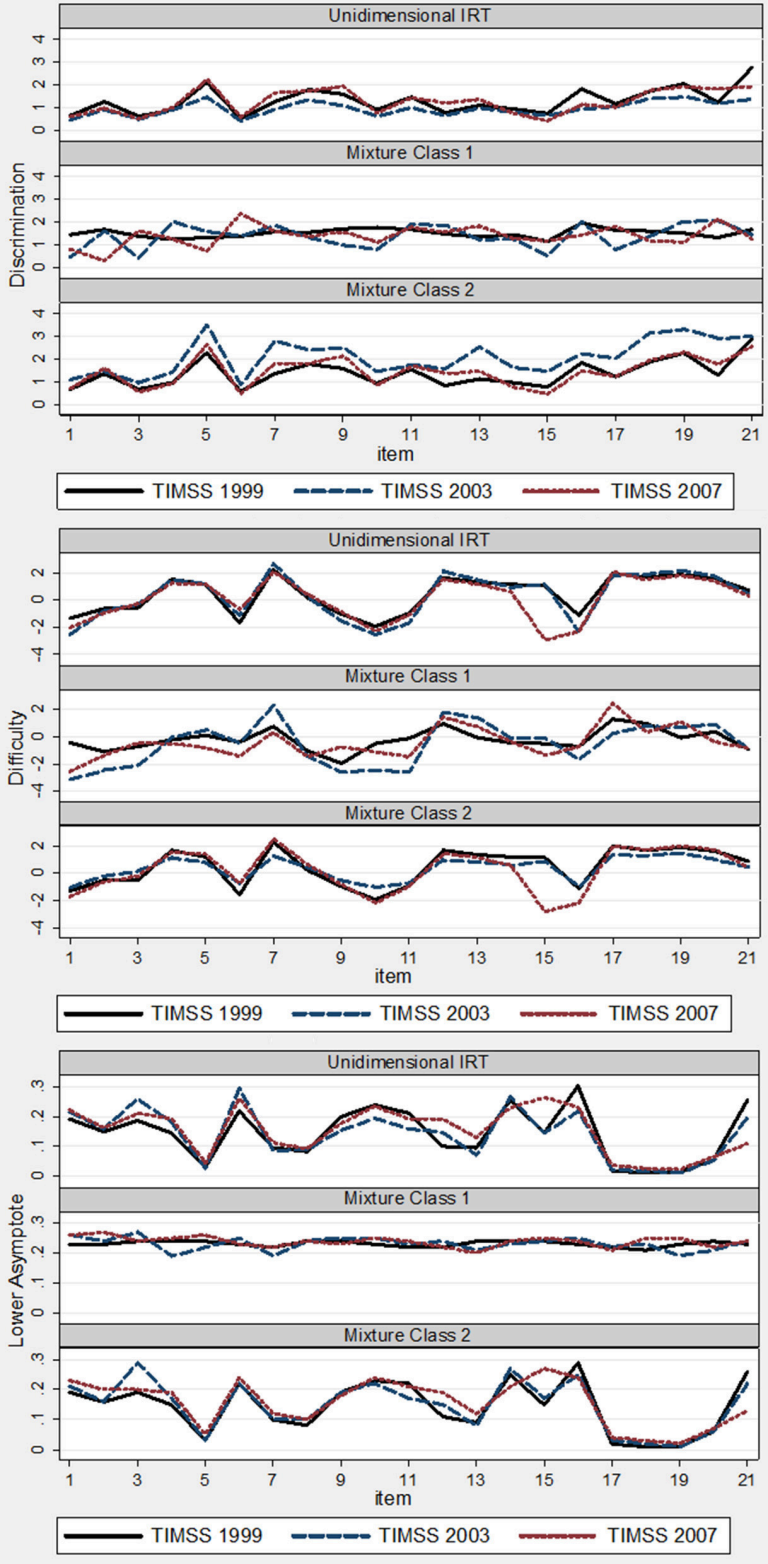
**Plots of item parameters by measurement model: TIMSS 1999, 2003, and 2007**.

**Figure 2:**
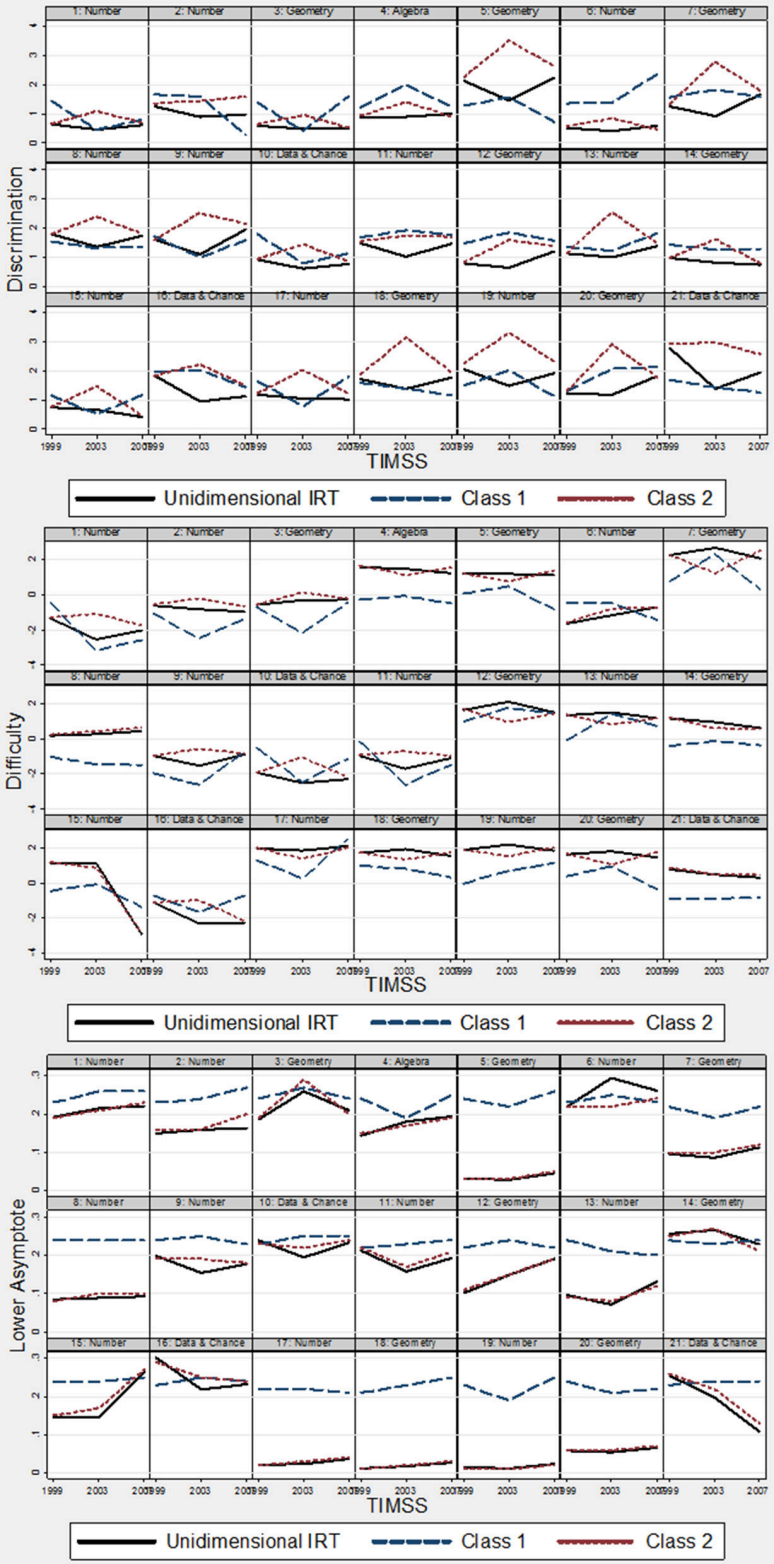
**Plots of changes in item parameters by item: TIMSS 1999, 2003, and 2007**.

Table 2 shows the results of the two-class mixture 3PL IRT model. To supplement understanding changes in item parameter estimates, two plots were created to illustrate changes in item parameters by measurement model (i.e., unidimensional versus mixture) and item. Figure 1 illustrates item parameters by measurement model. Figure 2 shows the changes in parameter values for the same item.

**Table 2:** **Item parameter estimates of the 21 trended anchor items in 1999, 2003, and 2007 TIMSS: Two-class mixture 3PL IRT model**.

**Class**	**Item**	**Discrimination (*****a*****)**			**Difficulty (*****b)***			**Lower asymptote (*****c*****)**		
		**1999**	**2003**	**2007**	**1999**	**2003**	**2007**	**1999**	**2003**	**2007**
1	1	1.44 (0.05)	0.46 (0.01)	0.80 (0.07)	−0.43 (0.13)	−3.16 (0.09)	−2.55 (0.12)	0.23 (0.00)	0.26 (0.00)	0.26 (0.00)
	2	1.68 (0.04)	1.61 (0.11)	0.30 (0.01)	−1.08 (0.13)	−2.48 (0.12)	−1.37 (0.12)	0.23 (0.00)	0.24 (0.00)	0.27 (0.00)
	3	1.39 (0.06)	0.42 (0.02)	1.61 (0.06)	−0.71 (0.12)	−2.15 (0.10)	−0.45 (0.06)	0.24 (0.00)	0.27 (0.01)	0.24 (0.00)
	4	1.24 (0.05)	2.01 (0.10)	1.26 (0.05)	−0.25 (0.10)	−0.06 (0.02)	−0.49 (0.07)	0.24 (0.00)	0.19 (0.01)	0.25 (0.00)
	5	1.30 (0.04)	1.58 (0.07)	0.73 (0.05)	0.08 (0.11)	0.50 (0.07)	−0.82 (0.10)	0.24 (0.00)	0.22 (0.01)	0.26 (0.00)
	6	1.38 (0.05)	1.40 (0.07)	2.37 (0.06)	−0.42 (0.13)	−0.47 (0.04)	−1.43 (0.06)	0.23 (0.00)	0.25 (0.01)	0.23 (0.00)
	7	1.57 (0.04)	1.84 (0.07)	1.59 (0.06)	0.75 (0.11)	2.32 (0.09)	0.33 (0.07)	0.22 (0.00)	0.19 (0.00)	0.22 (0.00)
	8	1.53 (0.06)	1.31 (0.06)	1.36 (0.06)	−1.03 (0.18)	−1.44 (0.08)	−1.48 (0.09)	0.24 (0.00)	0.24 (0.00)	0.24 (0.00)
	9	1.71 (0.04)	0.99 (0.04)	1.57 (0.06)	−1.96 (0.11)	−2.62 (0.11)	−0.74 (0.10)	0.24 (0.00)	0.25 (0.00)	0.23 (0.00)
	10	1.78 (0.04)	0.78 (0.05)	1.12 (0.07)	−0.51 (0.15)	−2.48 (0.12)	−1.13 (0.10)	0.23 (0.00)	0.25 (0.01)	0.25 (0.00)
	11	1.68 (0.06)	1.91 (0.12)	1.76 (0.07)	−0.15 (0.18)	−2.62 (0.10)	−1.48 (0.11)	0.22 (0.00)	0.23 (0.00)	0.24 (0.00)
	12	1.47 (0.05)	1.85 (0.07)	1.56 (0.05)	0.98 (0.11)	1.77 (0.10)	1.45 (0.10)	0.22 (0.00)	0.24 (0.01)	0.22 (0.00)
	13	1.34 (0.05)	1.22 (0.06)	1.82 (0.06)	−0.09 (0.11)	1.42 (0.12)	0.72 (0.11)	0.24 (0.00)	0.21 (0.01)	0.20 (0.00)
	14	1.43 (0.04)	1.25 (0.07)	1.28 (0.06)	−0.43 (0.10)	−0.12 (0.07)	−0.37 (0.10)	0.24 (0.00)	0.23 (0.01)	0.24 (0.00)
	15	1.15 (0.04)	0.52 (0.04)	1.17 (0.06)	−0.48 (0.08)	−0.09 (0.10)	−1.37 (0.10)	0.24 (0.00)	0.24 (0.01)	0.25 (0.00)
	16	1.97 (0.05)	2.03 (0.12)	1.44 (0.06)	−0.74 (0.12)	−1.67 (0.10)	−0.74 (0.15)	0.23 (0.00)	0.25 (0.01)	0.24 (0.00)
	17	1.65 (0.04)	0.79 (0.06)	1.79 (0.04)	1.27 (0.11)	0.25 (0.07)	20.46 (0.06)	0.22 (0.00)	0.22 (0.01)	0.21 (0.00)
	18	1.59 (0.05)	1.38 (0.07)	1.17 (0.06)	0.97 (0.11)	0.78 (0.06)	0.30 (0.10)	0.21 (0.00)	0.23 (0.01)	0.25 (0.00)
	19	1.50 (0.07)	2.02 (0.07)	1.12 (0.08)	−0.05 (0.19)	0.67 (0.04)	1.12 (0.09)	0.23 (0.00)	0.19 (0.00)	0.25 (0.00)
	20	1.32 (0.04)	2.06 (0.09)	2.13 (0.05)	0.37 (0.13)	0.92 (0.05)	−0.38 (0.07)	0.24 (0.00)	0.21 (0.01)	0.22 (0.00)
	21	1.68 (0.05)	1.44 (0.06)	1.26 (0.05)	−0.92 (0.16)	−0.91 (0.05)	−0.84 (0.07)	0.23 (0.00)	0.24 (0.01)	0.24 (0.00)
2	1	0.67 (0.01)	1.10 (0.03)	0.71 (0.02)	−1.29 (0.03)	−1.06 (0.03)	−1.72 (0.04)	0.19 (0.00)	0.21 (0.01)	0.23 (0.00)
	2	1.37 (0.05)	1.46 (0.02)	1.60 (0.05)	−0.54 (0.02)	−0.20 (0.02)	−0.65 (0.02)	0.16 (0.00)	0.16 (0.00)	0.20 (0.00)
	3	0.66 (0.02)	0.97 (0.02)	0.55 (0.01)	−0.55 (0.03)	0.14 (0.04)	−0.21 (0.03)	0.19 (0.00)	0.29 (0.01)	0.20 (0.00)
	4	0.95 (0.02)	1.41 (0.03)	0.92 (0.02)	1.67 (0.02)	1.15 (0.03)	1.55 (0.03)	0.15 (0.00)	0.17 (0.00)	0.19 (0.00)
	5	2.27 (0.06)	3.52 (0.06)	2.64 (0.05)	1.22 (0.01)	0.77 (0.01)	1.39 (0.01)	0.03 (0.00)	0.03 (0.00)	0.05 (0.00)
	6	0.58 (0.02)	0.86 (0.02)	0.47 (0.01)	−1.58 (0.05)	−0.76 (0.03)	−0.74 (0.05)	0.22 (0.01)	0.22 (0.01)	0.24 (0.01)
	7	1.35 (0.04)	2.80 (0.08)	1.80 (0.05)	2.30 (0.03)	1.25 (0.01)	2.54 (0.03)	0.10 (0.00)	0.10 (0.00)	0.12 (0.00)
	8	1.79 (0.02)	2.39 (0.03)	1.80 (0.04)	0.24 (0.02)	0.42 (0.01)	0.64 (0.01)	0.08 (0.00)	0.10 (0.00)	0.10 (0.00)
	9	1.59 (0.03)	2.51 (0.06)	2.14 (0.06)	−0.97 (0.02)	−0.56 (0.02)	−0.84 (0.02)	0.19 (0.00)	0.19 (0.01)	0.18 (0.00)
	10	0.93 (0.02)	1.44 (0.03)	0.86 (0.02)	−1.93 (0.04)	−1.05 (0.03)	−2.18 (0.04)	0.23 (0.00)	0.22 (0.01)	0.24 (0.00)
	11	1.54 (0.03)	1.74 (0.02)	1.69 (0.06)	−0.92 (0.03)	−0.71 (0.02)	−0.96 (0.03)	0.22 (0.01)	0.17 (0.01)	0.21 (0.01)
	12	0.82 (0.02)	1.58 (0.05)	1.37 (0.05)	1.70 (0.03)	0.96 (0.02)	1.44 (0.03)	0.11 (0.00)	0.15 (0.00)	0.19 (0.00)
	13	1.14 (0.03)	2.54 (0.04)	1.46 (0.04)	1.36 (0.02)	0.82 (0.01)	1.16 (0.02)	0.09 (0.00)	0.08 (0.00)	0.12 (0.00)
	14	0.97 (0.03)	1.61 (0.04)	0.78 (0.03)	1.20 (0.02)	0.60 (0.02)	0.55 (0.03)	0.25 (0.00)	0.27 (0.00)	0.21 (0.00)
	15	0.76 (0.02)	1.47 (0.04)	0.46 (0.01)	1.14 (0.02)	0.85 (0.02)	−2.83 (0.07)	0.15 (0.00)	0.17 (0.00)	0.27 (0.00)
	16	1.83 (0.06)	2.23 (0.05)	1.49 (0.04)	−1.14 (0.04)	−1.00 (0.02)	−2.18 (0.04)	0.29 (0.01)	0.25 (0.01)	0.24 (0.01)
	17	1.23 (0.03)	2.03 (0.03)	1.24 (0.04)	1.99 (0.03)	1.35 (0.02)	1.98 (0.04)	0.02 (0.00)	0.03 (0.00)	0.04 (0.00)
	18	1.89 (0.06)	3.15 (0.07)	1.95 (0.05)	1.70 (0.02)	1.31 (0.02)	1.73 (0.02)	0.01 (0.00)	0.02 (0.00)	0.03 (0.00)
	19	2.26 (0.07)	3.31 (0.06)	2.31 (0.06)	1.88 (0.02)	1.49 (0.02)	2.01 (0.02)	0.01 (0.00)	0.01 (0.00)	0.02 (0.00)
	20	1.30 (0.03)	2.91 (0.07)	1.77 (0.04)	1.65 (0.02)	1.02 (0.01)	1.74 (0.02)	0.06 (0.00)	0.06 (0.00)	0.07 (0.00)
	21	2.92 (0.05)	2.99 (0.06)	2.56 (0.06)	0.85 (0.02)	0.46 (0.01)	0.43 (0.01)	0.26 (0.00)	0.22 (0.00)	0.13 (0.00)

*Estimated using MCMC with 10,000 samples and 4000 samples burn-in; values in parenthesis represent MC Error*.

Similar to results in the unidimensional case, there were minimal deviations in item parameter estimates for the pseudo-guessing parameter; however, there were changes in both difficulty and discrimination. For item discrimination in latent class 1, the mean absolute deviations between 1999 and 2003 and between 2003 and 2007 were 0.45 and 0.54, respectively. The overall mean absolute deviation was 0.40. There were items with discrimination estimates that decreased by about 1; for example, between 1999 and 2003, item discrimination decreased by about 1 for items 1 and 3. On the other hand, for item 6, item discrimination increased by about 1 between 2003 and 2007. For latent class 2, there was an overall average absolute deviation of 0.72 between 1999 and 2003, and an average absolute deviation of 0.65 between 2003 and 2007. In general, the discrimination parameters for latent class 2 increased and then decreased across the three administrations.

For the difficulty parameter, the average absolute deviation for latent class 1 was about 1 between 1999 and 2003 and again about 1 between 2003 and 2007. However, for class 2, the average absolute deviation was about 0.50 between 1999 and 2003 and about 0.65 between 2003 and 2007. This shows that there was greater IPD in difficulty for latent class 1 than latent class 2.

#### Model fit

Table 3 shows the fit statistics of the unidimensional 3PL IRT model to the two-class mixture 3PL IRT model. For all three testing cycles, the AIC selected the two-class mixture 3PL IRT model over the unidimensional IRT model; the BIC selected the mixture model for 2003 and 2007. This provided evidence that the mixture model was a better fitting model than the unidimensional 3PL IRT model—and shows evidence of latent subgroups that are not invariant with respect to item parameters.

**Table 3:** **Fit statistics for the 21 trended anchor items in 1999, 2003, and 2007 TIMSS**.

**TIMSS**	**Unidimensional 3PL IRT model**			**2-Class 3PL mixture model**		
	**−2*LL***	***AIC***	***BIC***	**−2*LL***	***AIC***	***BIC***
1999	44,560	44,686	45,047	44,150	44,402	45,125
2003	89,510	89,636	90,040	87,980	88,232	89,039
2007	41,290	41,416	41,773	40,310	40,562	41,275

*Estimated using MCMC with 10,000 samples and 4000 samples burn-in. The −2LL is the posterior mean of the MCMC deviance (Li et al., 2009)*.

#### Latent class size and ability variance

Table 4 shows the class sizes and the variance estimates for ability. The size of latent class 1 in 1999 was 0.04; this increased to 0.19 in 2003, but decreased to 0.10 in 2007. Although there seemed to be two classes throughout the three administrations, a larger proportion of the examinees were classified to class 2 rather than class 1. The results of the variance estimates of examinee ability showed that they drifted from 2.27 in 1999 to 0.74 in 2003 and finally to 1.76 in 2007.

**Table 4:** **Class size for the 21 trended anchor items in 1999, 2003, and 2007 TIMSS using two-class mixture 3PL IRT model**.

	**1999**	**2003**	**2007**
Class 1	0.04 (0.01)	0.19 (0.01)	0.10 (0.01)
Class 2	0.96 (0.01)	0.81 (0.01)	0.90 (0.01)
*Var*(θ)	2.27 (0.07)	0.74 (0.01)	1.76 (0.05)

*Values in parenthesis represent MC Error*.

## Study 2: a simulation study

### Methods

The simulation study examined the impact of fitting data generated from a mixture IRT model with IPD and estimating the data using a unidimensional IRT model. The motivation for the simulation study was to investigate consequences of ignoring to check for invariance in latent subgroups, particularly with respect to parameter and ability estimates; and as such, the purpose of the simulation studies was to examine the magnitude of potential bias that could occur when such invariance properties are ignored. As demonstrated in the real-world study, evidence of latent subgroups existed in the TIMSS data; however, data continued to be calibrated ignoring the presence of latent subgroups. The mixture 2PL IRT model was employed to generate data. The mixture 2PL IRT model was used, as the results in Study 1 showed IPD in item discrimination and difficulty parameters, but not in the pseudo-guessing parameter. Estimated item parameters from 20 items of the TIMSS 1999 data were used as generating values to simulate data; item parameter values were obtained from the two-class mixture IRT model estimated from the real-world data analysis in Study 1. For the simulation setting, IPD in item difficulty and item discrimination were considered for two testing occasions and two latent subgroups for 20 items and 2000 examinees. The number of items was set to 20 for the simulation study, as the TIMSS released 23 items in its TIMSS 2003 database for 1995, 1999, and 2003 comparisons; in the TIMSS 2007 database, 21 items were released corresponding to trended items used in 1999, 2003, and 2007 administrations.

The simulation study considered five conditions, which are presented in Table 5. Two latent groups and two testing administrations were used. The generating values for latent class sizes were specified as 0.60 for Group 1 and 0.40 for Group 2, resembling class sizes used in Cohen and Bolt (2005). The generating values for parameter estimates used in Time 1 for both Groups 1 and 2 were identical for all five conditions; they served as a baseline comparison for changes in item parameters. Generating values for IPD were specified in Time 2 and were restricted to examinees in Group 1. This assumed a level of invariance for item parameters of examinees in Group 2.

**Table 5:** **Conditions for the simulation study: generating values**.

**Condition**	**Item**	**Time 1**				**Time 2**			
		**Group 1**		**Group 2**		**Group 1**		**Group 2**	
		***a*_11_**	***b*_11_**	***a*_12_**	***b*_12_**	***a*_21_**	***b***_**21**_	***a***_**22**_	***b***_**22**_
1 (Increase in Item Difficulty for Group 1 at Time 2)	1	1.44	−0.43	0.67	−1.29	1.44	0.57	0.67	−1.29
	2	1.68	−1.08	1.37	−0.54	1.68	−0.08	1.37	−0.54
	3	1.39	−0.71	0.66	−0.55	1.39	0.29	0.66	−0.55
	4	1.24	−0.25	0.95	1.67	1.24	0.75	0.95	1.67
	5	1.30	0.08	2.27	1.22	1.30	1.08	2.27	1.22
	6	1.38	−0.42	0.58	−1.58	1.38	0.58	0.58	−1.58
	7	1.57	0.75	1.35	2.30	1.57	1.75	1.35	2.30
	8	1.53	−1.03	1.79	0.24	1.53	−0.03	1.79	0.24
	9	1.71	−1.96	1.59	−0.97	1.71	−0.96	1.59	−0.97
	10	1.78	−0.51	0.93	−1.93	1.78	0.49	0.93	−1.93
	11	1.68	−0.15	1.54	−0.92	1.68	0.85	1.54	−0.92
	12	1.47	0.98	0.82	1.70	1.47	1.98	0.82	1.70
	13	1.34	−0.09	1.14	1.36	1.34	0.91	1.14	1.36
	14	1.43	−0.43	0.97	1.20	1.43	0.57	0.97	1.20
	15	1.15	−0.48	0.76	1.14	1.15	0.52	0.76	1.14
	16	1.97	−0.74	1.83	−1.14	1.97	0.26	1.83	−1.14
	17	1.65	1.27	1.23	1.99	1.65	2.27	1.23	1.99
	18	1.59	0.97	1.89	1.70	1.59	1.97	1.89	1.70
	19	1.50	−0.05	2.26	1.88	1.50	0.95	2.26	1.88
	20	1.32	0.37	1.30	1.65	1.32	1.37	1.30	1.65
2 (Increase in Item Discrimination for Group 1 atTime 2)	1	1.44	−0.43	0.67	−1.29	2.44	−0.43	0.67	−1.29
	2	1.68	−1.08	1.37	−0.54	2.68	−1.08	1.37	−0.54
	3	1.39	−0.71	0.66	−0.55	2.39	−0.71	0.66	−0.55
	4	1.24	−0.25	0.95	1.67	2.24	−0.25	0.95	1.67
	5	1.30	0.08	2.27	1.22	2.30	0.08	2.27	1.22
	6	1.38	−0.42	0.58	−1.58	2.38	−0.42	0.58	−1.58
	7	1.57	0.75	1.35	2.30	2.57	0.75	1.35	2.30
	8	1.53	−1.03	1.79	0.24	2.53	−1.03	1.79	0.24
	9	1.71	−1.96	1.59	−0.97	2.71	−1.96	1.59	−0.97
	10	1.78	−0.51	0.93	−1.93	2.78	−0.51	0.93	−1.93
	11	1.68	−0.15	1.54	−0.92	2.68	−0.15	1.54	−0.92
	12	1.47	0.98	0.82	1.70	2.47	0.98	0.82	1.70
	13	1.34	−0.09	1.14	1.36	2.34	−0.09	1.14	1.36
	14	1.43	−0.43	0.97	1.20	2.43	−0.43	0.97	1.20
	15	1.15	−0.48	0.76	1.14	2.15	−0.48	0.76	1.14
	16	1.97	−0.74	1.83	−1.14	2.97	−0.74	1.83	−1.14
	17	1.65	1.27	1.23	1.99	2.65	1.27	1.23	1.99
	18	1.59	0.97	1.89	1.70	2.59	0.97	1.89	1.70
	19	1.50	−0.05	2.26	1.88	2.50	−0.05	2.26	1.88
	20	1.32	0.37	1.30	1.65	2.32	0.37	1.30	1.65
3 (Increase in both Item Difficulty and Discrimination for Group 1 at Time 2)	1	1.44	−0.43	0.67	−1.29	2.44	0.57	0.67	−1.29
	2	1.68	−1.08	1.37	−0.54	2.68	−0.08	1.37	−0.54
	3	1.39	−0.71	0.66	−0.55	2.39	0.29	0.66	−0.55
	4	1.24	−0.25	0.95	1.67	2.24	0.75	0.95	1.67
	5	1.30	0.08	2.27	1.22	2.30	1.08	2.27	1.22
	6	1.38	−0.42	0.58	−1.58	2.38	0.58	0.58	−1.58
	7	1.57	0.75	1.35	2.30	2.57	1.75	1.35	2.30
	8	1.53	−1.03	1.79	0.24	2.53	−0.03	1.79	0.24
	9	1.71	−1.96	1.59	−0.97	2.71	−0.96	1.59	−0.97
	10	1.78	−0.51	0.93	−1.93	2.78	0.49	0.93	−1.93
	11	1.68	−0.15	1.54	−0.92	2.68	0.85	1.54	−0.92
	12	1.47	0.98	0.82	1.70	2.47	1.98	0.82	1.70
	13	1.34	−0.09	1.14	1.36	2.34	0.91	1.14	1.36
	14	1.43	−0.43	0.97	1.20	2.43	0.57	0.97	1.20
	15	1.15	−0.48	0.76	1.14	2.15	0.52	0.76	1.14
	16	1.97	−0.74	1.83	−1.14	2.97	0.26	1.83	−1.14
	17	1.65	1.27	1.23	1.99	2.65	2.27	1.23	1.99
	18	1.59	0.97	1.89	1.70	2.59	1.97	1.89	1.70
	19	1.50	−0.05	2.26	1.88	2.50	0.95	2.26	1.88
	20	1.32	0.37	1.30	1.65	2.32	1.37	1.30	1.65

*1. Generating values for Group 1 and Group 2 for Time 1 were obtained from estimated item parameters using the mixture 3PL IRT model of TIMSS 1999 data (items 1 to 20)*.

*2. All simulations in Conditions 1–3 used ability as normally distributed with mean 0 and variance 1, θ ~N(0,1)*.

3. In addition to the conditions listed above two additional conditions were examined:

Condition 4: Change in ability distribution for one group at Time 2

- Time 1: Same generating values used at Time 1 of Condition 1

- Time 2: Same generating values used at Time 1 of Condition 1 with θ ~N(1,0.5) for Group 1

Condition 5: Change in ability distribution for one group at Time 2 and increased discrimination

- Time 1: Same generating values used at Time 1 of Condition 1

*- Time 2: Same generating values used at Time 2 of Condition 2 (increase in discrimination for Group 1) with θ ~N(1,0.5)*.

In condition 1, generating values of item difficulty for examinees in Group 1 increased by 1 unit from Time 1 to Time 2; all other conditions remained constant. In condition 2, generating values for item discrimination in Group 1 increased by 1 unit, and in condition 3, both item difficulty and item discrimination in Group 1 increased by 1 unit. For all three conditions, examinee ability was assumed to be normally distributed with mean 0 and variance 1. However, in condition 4, the distribution of examinee ability was changed to mean 1 and variance of 0.50 for examinees in Group 1 of Time 2; there were no changes in generating values for this condition. Finally, in condition 5, examinees in Group 1 of Time 2 had an increase in generating values of item discrimination by 1 unit as well as ability distribution of mean 1 and variance 0.50. These different settings were created to reflect conditions from the real-world data results found in Study 1.

To examine the change in ability estimates between the population and the estimated values, root mean squared errors (RMSEs) were calculated as follows (Wells et al., 2002):

$$\begin{array}{l} RMSE_{r}=\sqrt{\frac{\overset{N}{\underset{i=1}{\sum }}{\left({\overset{\wedge }{\theta }}_{it}-\theta_{it}\right)}^{2}}{N}}. \end{array}$$

Here, *r* represents the specific replication, *N* is the number of examinees, and ${\overset{\wedge }{\theta }}_{it}$ is the estimate of θ for examinee *i* at time *t*. This statistic examined the change in examinee ability estimates compared to the generated ability parameters.

To examine the changes in examinee ability between the two testing administrations, root mean squared differences (RMSDs) were calculated (Wells et al., 2002):

$$\begin{array}{l} RMSD_{r}=\sqrt{\frac{\overset{N}{\underset{i=1}{\sum }}{\left({\overset{\wedge }{\theta }}_{i1}-{\overset{\wedge }{\theta }}_{i2}\right)}^{2}}{N}}. \end{array}$$

Changes in item parameters between the two testing occasions were measured by calculating bias between mean parameter estimates of Time 1 and Time 2.

Another aim of the simulation study was to investigate the effect of IPD on classification accuracy of the mixture IRT model. In other words, the quality of classification for deviation in item parameter was studied. This allows one to examine the effect of different patterns in IPD on the classification accuracy in a mixture IRT model; this statistic can also serve as a measure of detecting DIF. To measure classification accuracy, the expected proportion of cases correctly classified (*P*_*c*_) was calculated for each replication of data as follows (Clogg, 1995):

$$\begin{array}{l} P_{c}=\underset{s}{\sum }\left[n_{s}\times \max\mathrm{Pr}\left(G|Y_{1},Y_{2},\ldots ,Y_{20}\right)\right]∕N. \end{array}$$

Here, *s* indicates the unique response patterns and *n*_*s*_ corresponds to the frequency of each response pattern. Furthermore, maxPr(*G*|*Y*_1_, *Y*_2_, …, *Y*_*J*_) is the maximum posterior probability across the latent classes for a given response pattern, and *N* is the total number of examinees. Changes in *P*_*c*_ for different conditions were calculated and compared. These statistics were used as relative measures for comparison.

Data used for the simulation study were generated using Stata using a user-written macro based on the five conditions specified in Table 5. For each condition, 100 data replications were generated and estimated using Latent Gold (Vermunt and Magidson, 2007) using a DOS batch file for both the two-class mixture 2PL IRT model and the unidimensional 2PL IRT model. Latent Gold uses an EM algorithm then switches to the Newton-Raphson iterative process to finalize the estimation process. Latent Gold resolves issues in label switching by imposing constraints on the order of class sizes (Vermunt and Magidson, 2016). To avoid boundary estimation problems that are often found in latent class models, posterior mode estimation was used (Galindo-Garre and Vermunt, 2006). Output for each result from the replicated data was summarized with appropriate statistics calculated using Stata.

### Results

#### Item parameters

Table 6 presents the results for condition 1, which represents an increase in item difficulty by 1 for Group 1 at Time 2. The last column in the table shows the mean deviation in the parameter estimates between Time 1 and Time 2. For item discrimination, there were no clear patterns of increase or decrease. For example, for item 4, item discrimination decreased by 0.42, while for item 16, the estimates increased by 0.57. It should be noted here that for condition 1, there were no changes to item discrimination. The overall average absolute deviation in item discrimination was 0.34; however, taking into consideration the positive and negative changes, the overall deviation was less than 0.01. For item difficulty, there was an overall increase of about 0.84 between the two testing administrations. The largest increase was for item 9 with a deviance of 1.10 units, while the lowest increase was for item 15, by 0.65 units.

**Table 6:** **Fitting data generated from mixture IRT to unidimensional 2PL IRT model: Condition 1 (Increase in item difficulty for Group 1 at Time 2)**.

**Item**	**Time 1**		**Time 2**		**Change**	
	***a*_1_**	***b*_1_**	***a*_2_**	***b*_2_**	***a*_1_ − *a*_2_**	***b*_1_ − *b*_2_**
1	0.94 (0.07)	−0.69 (0.06)	1.27 (0.08)	0.06 (0.06)	−0.33	−0.75
2	1.65 (0.10)	−1.38 (0.08)	1.60 (0.10)	−0.38 (0.07)	0.05	−1.00
3	1.09 (0.07)	−0.71 (0.06)	1.18 (0.08)	0.05 (0.06)	−0.09	−0.76
4	1.38 (0.08)	0.44 (0.06)	0.97 (0.07)	1.13 (0.06)	0.42	−0.69
5	1.71 (0.10)	0.88 (0.07)	1.30 (0.09)	1.65 (0.08)	0.41	−0.77
6	0.85 (0.07)	−0.68 (0.05)	1.20 (0.08)	0.03 (0.06)	−0.35	−0.71
7	1.76 (0.11)	1.93 (0.10)	1.41 (0.12)	2.81 (0.13)	0.35	−0.88
8	1.79 (0.11)	−0.81 (0.08)	1.34 (0.08)	0.11 (0.06)	0.45	−0.91
9	1.83 (0.14)	−2.59 (0.13)	1.42 (0.10)	−1.49 (0.08)	0.41	−1.10
10	0.99 (0.07)	−1.12 (0.06)	1.54 (0.09)	−0.27 (0.06)	−0.55	−0.84
11	1.03 (0.07)	−0.60 (0.06)	1.59 (0.10)	0.24 (0.07)	−0.56	−0.84
12	1.09 (0.08)	1.37 (0.07)	1.19 (0.09)	2.16 (0.09)	−0.10	−0.79
13	1.48 (0.09)	0.54 (0.06)	1.15 (0.08)	1.30 (0.07)	0.33	−0.76
14	1.50 (0.09)	0.13 (0.06)	1.11 (0.08)	0.91 (0.06)	0.39	−0.78
15	1.19 (0.07)	0.04 (0.06)	0.91 (0.07)	0.69 (0.06)	0.28	−0.65
16	1.47 (0.10)	−1.50 (0.08)	2.04 (0.12)	−0.49 (0.08)	−0.57	−1.01
17	1.40 (0.10)	2.19 (0.10)	1.46 (0.13)	3.11 (0.14)	−0.06	−0.92
18	1.72 (0.11)	2.09 (0.10)	1.67 (0.14)	3.13 (0.15)	0.05	−1.05
19	1.86 (0.11)	1.06 (0.08)	1.17 (0.09)	1.83 (0.08)	0.68	−0.77
20	1.50 (0.09)	1.12 (0.07)	1.22 (0.09)	1.88 (0.09)	0.28	−0.76

*Simulated with 100 replications using posterior mode estimation. Values in parenthesis represent standard errors*.

Table 7 shows the results for condition 2. For this condition, item discrimination increased by 1 for examinees in Group 1 at Time 2. On average, changes in item discrimination increased item discrimination by about 0.51. Item difficulty was also affected; the average absolute change was 0.28. In general, results from conditions 1 and 2 indicated that an increase in either item discrimination or item difficulty directly affect and generally increased the manipulated parameter. The results also showed that these changes also shifted parameter estimates for the other non-manipulated parameter. That is, an increase in item difficulty also affected item discrimination and vice versa.

**Table 7:** **Fitting data generated from mixture IRT to unidimensional 2PL IRT model: Condition 2 (Increase in item discrimination for Group 1 at Time 2)**.

**Item**	**Time 1**		**Time 2**		**Change**	
	***a*_1_**	***b*_1_**	***a*_2_**	***b*_2_**	***a*_1_ − *a*_2_**	***b*_1_ − *b*_2_**
1	0.94 (0.07)	−0.69 (0.06)	1.48 (0.09)	−0.94 (0.07)	−0.55	0.25
2	1.65 (0.10)	−1.38 (0.08)	2.20 (0.14)	−1.86 (0.11)	−0.55	0.48
3	1.09 (0.07)	−0.71 (0.06)	1.65 (0.10)	−1.02 (0.07)	−0.57	0.32
4	1.38 (0.08)	0.44 (0.06)	2.02 (0.11)	0.41 (0.07)	−0.63	0.03
5	1.71 (0.10)	0.88 (0.07)	2.47 (0.14)	1.06 (0.09)	−0.75	−0.18
6	0.85 (0.07)	−0.68 (0.05)	1.40 (0.09)	−0.93 (0.07)	−0.55	0.25
7	1.76 (0.11)	1.93 (0.10)	2.27 (0.14)	2.48 (0.13)	−0.52	−0.55
8	1.79 (0.11)	−0.81 (0.08)	2.21 (0.13)	−1.16 (0.09)	−0.42	0.35
9	1.83 (0.14)	−2.59 (0.13)	1.76 (0.13)	−2.76 (0.13)	0.07	0.17
10	0.99 (0.07)	−1.12 (0.06)	1.59 (0.10)	−1.46 (0.08)	−0.61	0.34
11	1.03 (0.07)	−0.60 (0.06)	1.52 (0.09)	−0.74 (0.07)	−0.49	0.14
12	1.09 (0.08)	1.37 (0.07)	1.26 (0.08)	1.63 (0.08)	−0.17	−0.26
13	1.48 (0.09)	0.54 (0.06)	2.13 (0.12)	0.58 (0.08)	−0.65	−0.03
14	1.50 (0.09)	0.13 (0.06)	2.06 (0.11)	0.00 (0.07)	−0.57	0.14
15	1.19 (0.07)	0.04 (0.06)	1.77 (0.10)	−0.14 (0.07)	−0.58	0.17
16	1.47 (0.10)	−1.50 (0.08)	2.28 (0.14)	−2.07 (0.12)	−0.81	0.57
17	1.40 (0.10)	2.19 (0.10)	1.52 (0.10)	2.54 (0.11)	−0.12	−0.35
18	1.72 (0.11)	2.09 (0.10)	2.03 (0.13)	2.55 (0.13)	−0.31	−0.46
19	1.86 (0.11)	1.06 (0.08)	2.64 (0.15)	1.24 (0.10)	−0.79	−0.18
20	1.50 (0.09)	1.12 (0.07)	2.06 (0.11)	1.41 (0.09)	−0.56	−0.29

*Simulated with 100 replications using posterior mode estimation. Values in parenthesis represent standard errors*.

Table 8 shows the results for condition 3, where both item discrimination and item difficulty were increased by 1 for examinees in Group 1 at Time 2. Results showed that on average, there was an absolute deviation in item discrimination by 0.50. However, for items 8 and 9, there was a decrease in item discrimination by 0.14 and 0.48, respectively, even though there was an increase in the population values of the mixture IRT model. For item difficulty, there was an overall increase by 1.21. As such, an increase in both parameter estimates also directly led to an increase in both item discrimination and difficulty (with the exception for items 8 and 9).

**Table 8:** **Fitting data generated from mixture IRT to unidimensional 2PL IRT model: Condition 3 (Increase in both item difficulty and discrimination for Group 1 at Time 2)**.

**Item**	**Time 1**		**Time 2**		**Change**	
	***a*_1_**	***b*_1_**	***a*_2_**	***b*_2_**	***a*_1_ − *a*_2_**	***b*_1_ − *b*_2_**
1	0.94 (0.07)	−0.69 (0.06)	1.82 (0.10)	0.23 (0.07)	−0.88	−0.92
2	1.65 (0.10)	−1.38 (0.08)	1.96 (0.11)	−0.42 (0.07)	−0.31	−0.96
3	1.09 (0.07)	−0.71 (0.06)	1.68 (0.09)	0.16 (0.07)	−0.59	−0.87
4	1.38 (0.08)	0.44 (0.06)	1.57 (0.10)	1.59 (0.08)	−0.19	−1.15
5	1.71 (0.10)	0.88 (0.07)	2.13 (0.14)	2.48 (0.13)	−0.41	−1.60
6	0.85 (0.07)	−0.68 (0.05)	1.75 (0.10)	0.20 (0.07)	−0.90	−0.88
7	1.76 (0.11)	1.93 (0.10)	2.09 (0.17)	3.88 (0.21)	−0.33	−1.95
8	1.79 (0.11)	−0.81 (0.08)	1.65 (0.09)	0.15 (0.07)	0.14	−0.96
9	1.83 (0.14)	−2.59 (0.13)	1.35 (0.09)	−1.62 (0.08)	0.48	−0.96
10	0.99 (0.07)	−1.12 (0.06)	2.18 (0.12)	−0.19 (0.08)	−1.19	−0.93
11	1.03 (0.07)	−0.60 (0.06)	2.12 (0.12)	0.48 (0.08)	−1.09	−1.08
12	1.09 (0.08)	1.37 (0.07)	1.37 (0.11)	2.56 (0.11)	−0.28	−1.20
13	1.48 (0.09)	0.54 (0.06)	1.79 (0.11)	1.84 (0.10)	−0.31	−1.29
14	1.50 (0.09)	0.13 (0.06)	1.64 (0.10)	1.24 (0.08)	−0.15	−1.11
15	1.19 (0.07)	0.04 (0.06)	1.44 (0.09)	0.98 (0.07)	−0.25	−0.94
16	1.47 (0.10)	−1.50 (0.08)	2.86 (0.17)	−0.48 (0.09)	−1.39	−1.02
17	1.40 (0.10)	2.19 (0.10)	1.58 (0.14)	3.53 (0.17)	−0.18	−1.34
18	1.72 (0.11)	2.09 (0.10)	2.12 (0.18)	3.97 (0.22)	−0.40	−1.89
19	1.86 (0.11)	1.06 (0.08)	1.92 (0.13)	2.58 (0.13)	−0.07	−1.52
20	1.50 (0.09)	1.12 (0.07)	1.93 (0.13)	2.74 (0.13)	−0.43	−1.62

*Simulated with 100 replications using posterior mode estimation. Values in parenthesis represent standard errors*.

Table 9 shows the results for condition 4, where the ability distribution for examinees in Group 1 was altered to mean 1 and variance 0.50 for Time 2. For all previous simulations, examinee ability was assumed to be normally distributed with mean 0 and variance 1. This condition examined the change when a subpopulation's ability increased with less variance. Between Time 1 and Time 2, there were no changes in the population values of item parameters. However, results indicated that on average, item discrimination had an absolute deviation of 0.34. For some items, the increase in item discrimination was as high as 1.16 (item 19). On the other hand, item difficulty decreased on average by 0.95; this reflects the adjustment created by higher examinee ability, which may have lowered item difficulty. As these results show, shifting the distribution of examinee ability using a mixture IRT model also affected item parameters in the unidimensional IRT model.

**Table 9:** **Fitting data generated from mixture IRT to unidimensional 2PL IRT model: Condition 4 [change in ability distribution for Group 1 at Time 2 to θ~*N*(1,0.5)]**.

**Item**	**Time 1**		**Time 2**		**Change**	
	***a*_1_**	***b*_1_**	***a*_2_**	***b*_2_**	***a*_1_ − *a*_2_**	***b*_1_ − *b*_2_**
1	0.94 (0.07)	−0.69 (0.06)	0.88 (0.07)	−1.50 (0.07)	0.05	0.81
2	1.65 (0.10)	−1.38 (0.08)	1.97 (0.12)	−2.47 (0.12)	−0.32	1.09
3	1.09 (0.07)	−0.71 (0.06)	1.24 (0.08)	−1.51 (0.08)	−0.15	0.80
4	1.38 (0.08)	0.44 (0.06)	1.94 (0.10)	−0.35 (0.07)	−0.56	0.79
5	1.71 (0.10)	0.88 (0.07)	2.23 (0.12)	0.00 (0.08)	−0.52	0.89
6	0.85 (0.07)	−0.68 (0.05)	0.78 (0.07)	−1.46 (0.07)	0.07	0.78
7	1.76 (0.11)	1.93 (0.10)	2.10 (0.12)	0.87 (0.08)	−0.35	1.05
8	1.79 (0.11)	−0.81 (0.08)	2.59 (0.15)	−1.95 (0.12)	−0.80	1.15
9	1.83 (0.14)	−2.59 (0.13)	2.44 (0.19)	−3.81 (0.22)	−0.61	1.22
10	0.99 (0.07)	−1.12 (0.06)	0.85 (0.08)	−2.13 (0.08)	0.14	1.02
11	1.03 (0.07)	−0.60 (0.06)	0.92 (0.07)	−1.53 (0.07)	0.11	0.94
12	1.09 (0.08)	1.37 (0.07)	1.12 (0.07)	0.54 (0.06)	−0.03	0.82
13	1.48 (0.09)	0.54 (0.06)	1.94 (0.10)	−0.34 (0.07)	−0.46	0.88
14	1.50 (0.09)	0.13 (0.06)	2.04 (0.11)	−0.79 (0.08)	−0.55	0.92
15	1.19 (0.07)	0.04 (0.06)	1.60 (0.09)	−0.69 (0.07)	−0.40	0.73
16	1.47 (0.10)	−1.50 (0.08)	1.47 (0.11)	−2.71 (0.12)	0.00	1.21
17	1.40 (0.10)	2.19 (0.10)	1.46 (0.09)	1.19 (0.07)	−0.06	1.00
18	1.72 (0.11)	2.09 (0.10)	1.90 (0.11)	1.02 (0.08)	−0.18	1.07
19	1.86 (0.11)	1.06 (0.08)	3.01 (0.18)	0.09 (0.10)	−1.16	0.97
20	1.50 (0.09)	1.12 (0.07)	1.84 (0.10)	0.26 (0.07)	−0.34	0.86

*Simulated with 100 replications using posterior mode estimation. Values in parenthesis represent standard errors*.

Finally, the results for condition 5 are presented in Table 10. This condition reflects an increase in item discrimination for examinee in Group 1 at Time 2. Moreover, these examinees also had ability distribution with mean 1 and variance 0.50. This change altered both item discrimination and item difficulty in opposite directions. Item discrimination increased on average by 0.95. There were items with high deviations in item discrimination of nearly 2; for example, item 8 increased by 1.87, and item 19 increased by 2.32. For item difficulty, estimates decreased by 1.53 on average, with a shift as large as 2.27 for item 16.

**Table 10:** **Fitting data generated from mixture IRT to unidimensional 2PL IRT model: Condition 5 [increase in item discrimination and change in ability distribution for group 1 at Time 2 to θ~*N*(1,0.5)]**.

**Item**	**Time 1**		**Time 2**		**Change**	
	***a*_1_**	***b*_1_**	***a*_2_**	***b*_2_**	***a*_1_ − *a*_2_**	***b*_1_ − *b*_2_**
1	0.94 (0.07)	−0.69 (0.06)	1.42 (0.10)	−2.13 (0.10)	−0.48	1.44
2	1.65 (0.10)	−1.38 (0.08)	2.75 (0.18)	−3.40 (0.19)	−1.10	2.01
3	1.09 (0.07)	−0.71 (0.06)	1.89 (0.12)	−2.26 (0.11)	−0.80	1.56
4	1.38 (0.08)	0.44 (0.06)	2.79 (0.15)	−1.06 (0.10)	−1.41	1.49
5	1.71 (0.10)	0.88 (0.07)	3.10 (0.17)	−0.60 (0.10)	−1.39	1.49
6	0.85 (0.07)	−0.68 (0.05)	1.31 (0.09)	−2.10 (0.09)	−0.46	1.42
7	1.76 (0.11)	1.93 (0.10)	2.54 (0.14)	0.83 (0.09)	−0.79	1.10
8	1.79 (0.11)	−0.81 (0.08)	3.66 (0.24)	−3.07 (0.20)	−1.87	2.26
9	1.83 (0.14)	−2.59 (0.13)	3.14 (0.26)	−4.65 (0.32)	−1.31	2.07
10	0.99 (0.07)	−1.12 (0.06)	1.38 (0.11)	−2.82 (0.13)	−0.39	1.70
11	1.03 (0.07)	−0.60 (0.06)	1.39 (0.09)	−2.09 (0.10)	−0.36	1.50
12	1.09 (0.08)	1.37 (0.07)	1.29 (0.07)	0.55 (0.06)	−0.21	0.82
13	1.48 (0.09)	0.54 (0.06)	2.69 (0.14)	−0.96 (0.10)	−1.21	1.50
14	1.50 (0.09)	0.13 (0.06)	2.85 (0.15)	−1.54 (0.11)	−1.35	1.67
15	1.19 (0.07)	0.04 (0.06)	2.38 (0.13)	−1.45 (0.10)	−1.19	1.49
16	1.47 (0.10)	−1.50 (0.08)	2.30 (0.18)	−3.77 (0.21)	−0.84	2.27
17	1.40 (0.10)	2.19 (0.10)	1.57 (0.09)	1.35 (0.08)	−0.17	0.84
18	1.72 (0.11)	2.09 (0.10)	2.13 (0.12)	1.06 (0.09)	−0.41	1.02
19	1.86 (0.11)	1.06 (0.08)	4.18 (0.25)	−0.61 (0.13)	−2.32	1.67
20	1.50 (0.09)	1.12 (0.07)	2.41 (0.12)	−0.07 (0.08)	−0.91	1.19

*Simulated with 100 replications using posterior mode estimation. Values in parenthesis represent standard errors*.

In summary, these changes in item parameters and examinee ability generated from a mixture IRT model showed that they affected parameter estimates when fit using a unidimensional IRT model, resulting in significant item parameter bias. In particular, changes involving only item difficulty or item discrimination affected both item parameters. Results also showed altering examinee ability affected both discrimination and difficulty parameters.

#### Examinee ability

To examine how changes in item parameters and in examinee ability distribution affected estimates for examinee ability, RMSE was calculated. Table 11 shows these results for the five conditions. Since, RMSE reflects the recovery of examinee ability, both mixture IRT and unidimensional IRT models were used. Both cases for Time 1 and Time 2 are presented. For Time 1 (same for all conditions), the RMSE was 0.21 for the mixture IRT model and 0.28 for the unidimensional IRT model. This shows that the mixture IRT model had a slightly lower RMSE than the unidimensional IRT model. This pattern was the same in Time 2 for conditions 1, 2, and 3. For an increase in item difficulty by 1, the RMSE decreased slightly for both models. However, the RMSE seemed to decrease more for the mixture IRT model when there was a shift involving the discrimination parameter (from 0.21 to 0.18). In conditions 4 and 5 that involve a change in ability distribution, RMSE increased to 0.74 for the mixture IRT model, representing an increase of about 0.50. RMSE also increased for the unidimensional IRT model to 0.61 by about 0.30. This shows that the mixture IRT model had greater bias for examinee ability when their distribution changed.

**Table 11:** **Changes in the root mean squared error (RMSE) of ability estimates using population and estimated values**.

**Condition**	**Model**	**RMSE**	
		**Time 1**	**Time 2**
1	Two-Class 2PL Mixture IRT	0.21	0.20
	Unidimensional 2PL IRT	0.28	0.24
2	Two-Class 2PL Mixture IRT	0.21	0.18
	Unidimensional 2PL IRT	0.28	0.26
3	Two-Class 2PL Mixture IRT	0.21	0.18
	Unidimensional 2PL IRT	0.28	0.27
4	Two-Class 2PL Mixture IRT	0.21	0.74
	Unidimensional 2PL IRT	0.28	0.61
5	Two-Class 2PL Mixture IRT	0.21	0.73
	Unidimensional 2PL IRT	0.28	0.63

*Simulated with 100 replications using posterior mode estimation. Values in parenthesis represent standard errors*.

Table 12 presents the RMSD that reflects the changes in examinee ability across the two scoring occasions. Overall, the RMSDs were relatively minor. However, there were patterns that emerged. For condition 1 that only involved changes in item difficulty, there was almost no change in RMSD. When discrimination increased, RMSD increased to 0.015 for condition 2, which was nearly twice the difference in the unidimensional RMSD of 0.008; a similar pattern was shown for condition 3, where both item discrimination and difficulty increased. For conditions 4 and 5, which involved changes in examinee ability distribution, the deviations were larger for the unidimensional IRT model by nearly double the RMSD of the mixture IRT model. For example in condition 5, the RMSD for the mixture IRT model was 0.012, while it was 0.021 for the unidimensional IRT model.

**Table 12:** **Changes in the root mean squared difference (RMSD) of ability using estimated ability across Time 1 and Time 2**.

**Condition**	**RMSD**	
	**Two-class 2PL mixture IRT**	**Unidimensional 2PL IRT**
1	0.003	0.002
2	0.015	0.008
3	0.014	0.007
4	0.003	0.006
5	0.012	0.021

*Simulated with 100 replications using posterior mode estimation. Values in parenthesis represent standard errors*.

#### Latent class sizes

Given the population values of 0.60 and 0.40 as the sizes for latent class 1 and latent class 2, respectively, the class sizes were well recovered, regardless of IPD when fit using the mixture IRT model. For conditions that involve an increase in item discrimination for examinees in Group 1, the class size was slightly overestimated. This was found for conditions 2, 3, and 5, which had size estimates of 0.601, 0.603, and 0.605, respectively. In general, IPD did not affect latent class sizes greatly.

#### Classification accuracy

To examine how changes in item parameters or examinee ability distribution increased classification accuracy, proportion correctly classified (*P*_*c*_) was calculated for each condition and is presented in Table 13. The *P*_*c*_ can be viewed as a statistic that measures DIF, as it relates to the quality of classification based on posterior probabilities, which determine whether an examinee belongs to a specific latent class. For a change in item difficulty, the *P*_*c*_ increased by only 0.005 (condition 1). However, for an increase in discrimination, the *P*_*c*_ increased by 0.028 (condition 2). The *P*_*c*_ also increased on average by about 0.042 when the distribution of examinee ability changed (conditions 4 and 5). Compared to item difficulty, changes in item discrimination had a greater impact on classification accuracy—this can affect the degree of DIF. IPD in a mixture IRT model resulting from item discrimination can lead to a minor, yet greater effect on DIF, than changes in item difficulty.

**Table 13:** **Classification accuracy: Proportion correctly classified**.

**Condition**	**Time 1**	**Time 2**
1	0.875	0.880
2	0.875	0.903
3	0.875	0.906
4	0.875	0.918
5	0.875	0.915

*Simulated with 100 replications using posterior mode estimation*.

## Discussion

This study examined the impact of IPD and the potentially compounded bias that may occur when the underlying baseline measurement model also holds mixture distributions. A motivation for conducting this study was that tests and measurement models that rely on assumptions of item invariance at baseline have solely been limited to checking for DIF based on manifest variables, while latent characteristics may be ignored. Real-world analysis showed evidence in the presence of mixture distributions using large-scale testing data, and the ensuing simulation study investigated the impact of bias on parameter and ability estimates. These findings call to caution and underscore the need to check for latent subgroups when using IRT models.

Results from real-world data in Study 1 showed that even within an assessment designed for unidimensional analysis, there were latent subgroups that support the analysis of IPD using a mixture IRT model. A real-world data analysis using the 1999, 2003, and 2007 TIMSS administrations showed that a two-class mixture 3PL IRT model fits the data better than a unidimensional 3PL IRT model. Based on estimated item parameters, there were minor deviations in the pseudo-guessing parameter. However, item discrimination and difficulty changed in a notable degree between the three testing administrations. Furthermore, the variance of examinee ability also changed. These findings provided grounds for further analysis using a simulation study.

The simulation study in Study 2 examined the effects of fitting data with latent subgroups using a unidimensional IRT model. Since there were only minor changes in the pseudo-guessing parameter, the two-class mixture 2PL model was used to generate data. Generating values were taken from the estimated item parameters in the TIMSS 1999 results for 20 items. Five conditions were simulated to examine the effect of item parameter estimates and examinee ability estimates when there was IPD resulting from a two-class mixture 2PL model.

Results showed that even when there was IPD resulting from item difficulty in one latent subgroup, this can lead to IPD in both difficulty and discrimination parameter estimates; a similar result was found when only item discrimination was altered. Simulations also showed that changing the distribution of examinee ability can lead to IPD in the unidimensional IRT model. To examine changes in examinee ability, IPD due to an increase in item discrimination affected examinee ability more than changes in item difficulty. The change in examinee ability distribution increased even further when both item discrimination and the distribution of examinee ability changed.

These findings shed new insights into the IPD literature, particularly on the need to check for invariance in latent subgroups. For operational testing programs, population homogeneity has to be examined for each test administration, meaning that the class structure needs to be checked at each time point. Previous studies have examined IPD as a different type of DIF, where the bias was between testing administrations. The results from this study showed that both DIF and IPD can occur, as evidenced by TIMSS 1999, 2003, and 2007 data. The 21 items that were used in this study were trended anchor items that were used to link scores between testing administrations. Although this study showed that the effect of IPD on examinee ability was minor, further investigation into this issue is warranted. Minimal changes in examinee ability arising from multiple factors—changes in item parameters and in examinee ability—can again affect model-based prediction of examinee ability. Moreover, statistics that facilitate detection of IPD for latent subgroups need to be developed with practically useful criteria for applied users.

There are a variety of factors that can lead to IPD. However, when researchers fail to check for the presence of latent subgroups, bias in item and ability estimates may increase even further when IPD occurs. Given the nature of the data, this study examined the behavior of trended anchor items that were used for linking and scaling test scores. This issue becomes critical for test development that use anchor items to trend ability scores. The investigation of IPD of mixture distributions should provide new understandings not only on traditional issues of DIF, but also on equating and linking mixture IRT models, which is beginning to raise interest among researchers (Paek et al., 2010; Han et al., 2012; Makransky et al., 2014). At the least, this study provides strong motivation for operational testing programs to check for invariance from latent characteristics at baseline, in addition to testing for DIF based on manifest variables to support the invariance assumption of IRT models. For greater generality, future simulation studies should include more varied conditions including multiple latent subgroups (more than 3 groups) as well as examining different item lengths and sample sizes. These conditions may provide a better understanding of IPD as well as their effect on large-scale assessments.

## Author contributions

YP, YL, and KX contributed to the conceptualization and design of the work, including the acquisition, analysis, and interpretation of data. YP, YL, and KX were involved in drafting and revising the manuscript. YP, YL, and KX approve the final manuscript submitted. YP, YL, and KX agree to be accountable for all aspects of the work, including accuracy and integrity.

### Conflict of interest statement

The authors declare that the research was conducted in the absence of any commercial or financial relationships that could be construed as a potential conflict of interest.

## References

[B1] Ackerman T. A. (1992). A didactic explanation of item bias, item impact, and item validity from a multidimensional perspective. J. Educ. Meas. 29, 67–91. 10.1111/j.1745-3984.1992.tb00368.x

[B2] Babcock B. Albano A. D. (2012). Rasch scale stability in the presence of item parameter and trait drift. Appl. Psychol. Meas. 36, 565–580. 10.1177/0146621612455090

[B3] Baker F. B. Kim S.-H. (2004). Item Response Theory: Parameter Estimation Techniques, 2nd Edn. New York, NY: Marcel Dekker.

[B4] Baucal A. Pavlovic-Babic D. Willms J. D. (2006). Differential selection into secondary schools in Serbia. Prospects Q. Rev. Comp. Educ. 36, 539–546. 10.1007/s11125-006-9011-9

[B5] Black P. Wiliam D. (2007). Large-scale assessment systems: design principles drawn from international comparisons. Meas. Interdiscip. Res. Perspect. 5, 1–53. 10.1080/15366360701293386

[B6] Bock R. Muraki E. Pfeiffenberger W. (1988). Item pool maintenance in the presence of item parameter drift. J. Educ. Meas. 25, 275–285. 10.1111/j.1745-3984.1988.tb00308.x

[B7] Bolt D. M. Cohen A. S. Wollack J. A. (2002). Item parameter estimation under conditions of test speededness: application of mixture Rasch model with ordinal constraints. J. Educ. Meas. 39, 331–348. 10.1111/j.1745-3984.2002.tb01146.x

[B8] Cho S.-J. Cohen A. S. (2010). A multilevel mixture IRT model with an application to DIF. J. Educ. Behav. Stat. 35, 336–370. 10.3102/1076998609353111

[B9] Cho S.-J. Cohen A. S. Kim S.-H. (2012). Markov chain Monte Carlo estimation of a mixture item response theory model. J. Stat. Comput. Simul. 83, 278–306. 10.1080/00949655.2011.603090

[B10] Choi Y.-J. Alexeev N. Cohen A. S. (2015). Differential item functioning analysis using a mixture 3-parameter logistic model with a covariate on the TIMSS 2007 mathematics test. Int. J. Test. 15, 239–253. 10.1080/15305058.2015.1007241

[B11] Clogg C. C. (1995). Latent class models, in Handbook of Statistical Modeling for the Social and Behavioral Sciences, eds Arminger G. Clogg C. C. Sobel M. E. (New York, NY: Plenum Press), 311–359.

[B12] Cohen A. S. Bolt D. M. (2005). A mixture model analysis of differential item functioning. J. Educ. Meas. 42, 133–148. 10.1111/j.1745-3984.2005.00007

[B13] Cohen A. S. Gregg N. Deng M. (2005). The role of extended time and item content on a high-stakes mathematics tests. Learn. Disabil. Res. Pract. 20, 225–233. 10.1111/j.1540-5826.2005.00138.x

[B14] DeMars C. E. Lau A. (2011). Differential item functioning detection with latent classes: how accurately detect who is responding differentially? Educ. Psychol. Meas. 71, 597–616. 10.1177/0013164411404221

[B15] Everitt B. S. Hand D. J. (1981). Finite Mixture Distribution. London: Chapman and Hall.

[B16] Galindo-Garre F. G. Vermunt J. K. (2006). Avoiding boundary estimates in latent class analysis by bayesian posterior mode estimation. Behaviormetrika 33, 43–59. 10.2333/bhmk.33.43

[B17] Gelman A. Rubin D. B. (1992). Inference from iterative simulation using multiple sequences. Stat. Sci. 7, 457–472. 10.1214/ss/1177011136

[B18] Goldstein H. (1983). Measuring changes in educational attainment over time: problems and possibilities. J. Educ. Meas. 20, 369–377. 10.1111/j.1745-3984.1983.tb00214.x

[B19] Hambleton R. K. Swaminathan H. (1985). Item Response Theory: Principles and Applications. Boston, MA: Kluwer-Nijhoff.

[B20] Hambleton R. K. Swaminathan H. Rogers J. (1991). Item Response Theory, Vol. 2 Hillsdale, NJ: Lawrence Erlbaum.

[B21] Han K. T. Wells C. S. Sireci S. G. (2012). The impact of multidirectional item parameter drift on IRT scaling coefficients and proficiency estimates. Appl. Meas. Educ. 25, 97–117. 10.1080/08957347.2012.660000

[B22] Hutchison D. Schagen I. (2007). Comparisons between PISA and TIMSS – are we the man with two watches, in Lessons Learned: What International Assessments Tell Us About Math Achievement, ed Loveless T. (Washington, DC: Brookings Institute Press), 227–262.

[B23] Juve J. A. (2004). Assessing Differential Item Functioning and Item Parameter Drift in the College Basic Academic Subjects Examination. Unpublished doctoral dissertation, University of Missouri, Columbia, MO.

[B24] Li F. Cohen A. S. Kim S.-H. Cho S.-J. (2009). Model selection methods for mixture dichotomous IRT models. Appl. Psychol. Meas. 33, 353–373. 10.1177/0146621608326422

[B25] Lord F. (1980). Applications of Item Response Theory to Practical Testing Problems. Hillsdale, NJ: Lawrence Erlbaum.

[B26] Lu R. Jiao H. (2009). Detecting DIF using the mixture Rasch model, in Paper Presented at the Annual Meeting of the National Council on Measurement in Education (San Diego, CA).

[B27] Lunn D. J. Thomas A. Best N. Spiegelhalter D. (2005). WinBUGS - a Bayesian modelling framework: concepts, structure, and extensibility. Stat. Comput. 10, 325–337. 10.1023/A:1008929526011

[B28] Maij-de Meij A. M. Kelderman H. van der Flier H. (2008). Fitting a mixture item response theory model to personality questionnaire data: characterizing latent classes and investigating possibilities for improving prediction. Appl. Psychol. Meas. 32, 611–631. 10.1177/0146621607312613

[B29] Makransky G. Schnohr C. W. Torsheim T. Currie C. (2014). Equating the HBSC family affluence scale across survey years: a method to account for item parameter drift using the Rasch model. Q. Life Res. 23, 2899–2907. 10.1007/s11136-014-0728-2 24902938

[B30] McLachlan G. Peel D. (2000). Finite Mixture Models. New York, NY: Wiley.

[B31] Miller G. E. Gesn P. R. Rotou J. (2005). Expected linking error resulting from item parameter drift among the common items on Rasch calibrated tests. J. Appl. Meas. 6, 48–56. 15701943

[B32] Mislevy R. J. (1982). Five steps toward controlling item parameter drift, in Paper Presented at the Annual Meeting of the American Educational Research Association (New York, NY).

[B33] Mislevy R. J. Johnson E. G. Muraki E. (1992). Chapter 3: scaling procedures in NAEP. J. Educ. Behav. Stat. 17, 131–154. 10.3102/10769986017002131

[B34] OECD (2014). PISA 2012 Technical Report. Paris: OECD Publishing.

[B35] Olson J. F. Martin M. O. Mullis I. V. S. (2009). TIMSS 2007 Technical Report. Chestnut Hill, MA: IEA.

[B36] Paek I. Cho S.-J. Cohen A. (2010). A comment on scale linking in mixture IRT modeling, in Paper Presented at the Annual Meeting of the National Council on Measurement in Education (Denver, CO).

[B37] Pleysier S. Pauwels L. Vervaeke G. Goethals J. (2005). Temporal invariance in repeated cross-sectional ‘Fear of Crime’ research. Int. Rev. Victimol. 12, 273–292. 10.1177/026975800501200304

[B38] Rost J. (1990). Rasch models in latent classes: an integration of two approaches to item analysis. Appl. Psychol. Meas. 14, 271–282. 10.1177/014662169001400305

[B39] Rost J. Carstensen C. von Davier M. (1997). Applying the mixed Rasch model to personality questionnaires, in Application of Latent Trait and Latent Class Models in the Social Sciences, eds Rost J. Langeheine R. (Munster: Waxmann), 324–332.

[B40] Rupp A. A. Zumbo D. B. (2006). Understanding parameter invariance in unidimensional IRT models. Educ. Psychol. Meas. 66, 63–84. 10.1177/0013164404273942

[B41] Samuelsen K. M. (2008). Examining differential item functioning from a latent mixture perspective, in Advances in Latent Variable Mixture Models, eds Hancock G. R. Samuelsen K. M. (Charlotte, NC: Information Age), 177–197.

[B42] Schmitt A. P. Holland P. Dorans N. J. (1993). Evaluating hypotheses about differential item functioning, in Differential Item Functioning, eds Holland P.W. Wainer H. (Hillsdale, NJ: Lawrence Erlbaum), 281–315.

[B43] Skykes R. C. Ito K. (1993). Item parameter drift in IRT-based licensure examinations, Paper Presented at the Annual Meeting of the National Council on Measurement in Education (Atlanta, GA).

[B44] Spiegelhalter D. J. Best N. G. Carlin B. P. van der Linde A. (2002). Bayesian measures of model complexity and fit. J. R. Stat. Soc. B 64, 583–639. 10.1111/1467-9868.00353

[B45] Stephens M. (2010). Dealing with label switching in mixture models. J. R. Stat. Soc. B 62, 795–809. 10.1111/1467-9868.00265

[B46] Takayama K. (2007). “A Nation at Risk” crosses the Pacific: transnational borrowing of the U.S. crisis discourse in the debate on education reform in Japan. Comp. Educ. Rev. 51, 423–446. 10.1086/520864

[B47] Titterington D. Smith A. Makov U. (1985). Statistical Analysis of Finite Mixture Distributions. New York, NY: Wiley.

[B48] Vermunt J. K. Magidson J. (2007). LG-SyntaxTM User's Guide: Manual for Latent Gold 4.5 Syntax Module. Belmont, MA: Statistical Innovations Inc.

[B49] Vermunt J. K. Magidson J. (2016). Technical Guide for Latent Gold 5.1: Basic, Advanced, and Syntax. Belmont, MA: Statistical Innovations, Inc.

[B50] von Davier M. Rost J. (1995). Mixture distribution Rasch models, in Rasch Models—Foundations, Recent Developments, and Applications, eds Fischer G. H. Molenaar I. W. (New York, NY: Springer), 257–268.

[B51] Wells C. S. Hambleton R. K. Kirkpatrick R. Meng Y. (2014). An examination of two procedures for identifying consequential item parameter drift. Appl. Meas. Educ. 27, 214–231. 10.1080/08957347.2014.905786

[B52] Wells C. S. Subkoviak M. J. Serlin R. C. (2002). The effect of item parameter drift on examinee ability estimates. Appl. Psychol. Meas. 26, 77–87. 10.1177/0146621602261005

[B53] Wollack J. A. Cohen A. S. Wells C. S. (2003). A method for maintaining scale stability in the presence of test speededness. J. Educ. Meas. 40, 307–330. 10.1111/j.1745-3984.2003.tb01149.x

[B54] Wu A. D. Li Z. Ng S. L. Zumbo B. D. (2006). Investigating and comparing the item parameter drift in the mathematics anchor/trend items in TIMSS between Singapore and the United States, in Paper Presented at the 32nd Annual Conference in International Association for Educational Assessment (Singapore).

